# Assessment of remote sensing-based indices for drought monitoring in the north-western region of Bangladesh

**DOI:** 10.1016/j.heliyon.2023.e13016

**Published:** 2023-01-21

**Authors:** Ashim C. Das, Shihab A. Shahriar, Md A. Chowdhury, Md Lokman Hossain, Shahed Mahmud, Md Kamruzzaman Tusar, Romel Ahmed, Mohammed Abdus Salam

**Affiliations:** a Department of Environmental Science and Disaster Management, Noakhali Science and Technology University, Noakhali, 3814, Bangladesh; b Department of Earth and Atmospheric Sciences University of Houston, TX, 77004, USA; c Department of Climate and Disaster Management, Jashore University of Science and Technology, Jashore, 7408, Bangladesh; d Department of Environment Protection Technology, German University Bangladesh, Gazipur, Bangladesh; e Department of Geography, Hong Kong Baptist University, Hong Kong, China; f Department of Forestry and Environmental Science, Shahjalal University of Science and Technology, Sylhet, 3114, Bangladesh

**Keywords:** Drought, Vegetation indices, Land use, Climate change, Remote sensing, Vegetation health, Natural hazard

## Abstract

Drought is a widespread hazard that can tremendously affect the biodiversity, habitat of wild species, and ecosystem functioning and stability, especially in the dry region. Due to its geographic location, the north-western region of Bangladesh has a comparatively arid climate which is very much susceptible to drought occurrence and is marked as a red zone. Despite the growing evidence of the impact of drought on food security and ecosystem functioning, little effort has been paid to mitigate the drought in this region. The present study aimed to assess the drought condition of the north-western region of Bangladesh using earth observation techniques. For this purpose, Landsat data from 1990 to 2020 was used to determine various vegetation indices such as Normalized Difference Vegetation Index (NDVI), Water Index (NDWI), Moisture Index (NDMI) and Soil Adjusted Vegetation Index (SAVI), along with Land Surface Temperature (LST). Results show that the depletion of forests (2832 km^2^) and water bodies (6773 km^2^) resulted from the expansion of settlement (6563 km^2^) and agricultural land (1802 km^2^) for the period 1990–2020. Examination of the temporal changes of vegetation indices and LST showed that the values of all indices decreased while the LST increased. The negative correlation between NDVI value and LST indicates that the vegetation in our study was subject to drought-induced shocks. This study reveals the current situation of the vegetation health in the north-western region of Bangladesh in relation to the drought conditions. The findings of this study have practical implications for the policymakers in implementing necessary measures for agriculture, forests, water development, and economic zone planning.

## Highlights

• Drought conditions in north-western region of Bangladesh were examined for 30 years.
• Depletion of forests and waterbodies resulted from the expansion of settlement.
• Vegetation greenness was negatively correlated with land surface temperature.
• Decreasing rate of vegetation indices value was higher in 2010–2020 than in 1990–2005.

## Graphical abstract

**Figure:**
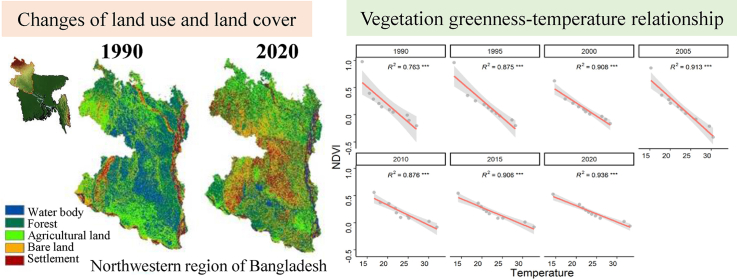

## 1 Introduction

Drought is one of the world's most destructive natural disasters, occurring at varying frequencies and intensities throughout a wide range of climatic areas [1,2]. Globally, drought has a substantial impact on food and water security [3], but its effects are contingent on the capacity to mitigate its social, economic, and environmental consequences [4]. Climate extremes such as periods of heavy rain and droughts will be more severe and widespread as a result of the increased likelihood and stringency of the warming climate pattern [5,6,7]. Although the changing pattern of climate has both positive and harmful effects on agriculture, it is more likely to exacerbate the drought scenarios [8]. Drought has been identified as one of the most severe extreme events, based on the number of people affected [9]. Between 1980 and 2008, the world saw around 410 serious droughts, affecting approximately 53.5 million people annually [9]. The increasing frequency and intensity of drought has affected several tropical regions in Southeast Asia, Africa, the northeastern half of Brazil, and Australia [10,11,12,13]. For example, droughts have plagued India since the mid-1990s with disastrous consequences. It is hurting the agricultural production as well as the economy from local to the national level since the majority of its people depend on agriculture [14,15].

Bangladesh is one of the most susceptible nations to natural disasters, particularly floods, cyclones, storm surges, droughts, heatwaves, sea level rise, and salinity intrusion due to its geographic location and socioeconomic status [16]. In recent decades, the country has been experiencing many types of natural disasters, including varying degree of droughts [17,18], which is affecting its socio-environmental dimensions [19]. Due to extremely low precipitation from November to May, Bangladesh is prone to drought, which can occur at any time of the year but is most common in the pre-monsoon (March–May) and post-monsoon seasons (October-November) [17,20]. The uneven distribution of precipitation across the country, particularly in the northern part of Bangladesh, accelerates the occurrences of devastating droughts, which has been recorded in several studies [21,22]. Since its independence, Bangladesh had experienced some exceptional droughts in 1973, 1978–79, 1981–82, 1989, 1992, 1994–95, 2000, 2006, 2009, 2012 and 2016 [22]. These droughts affected natural resources in the country, particularly, forests, fisheries, agricultural production and other industries that depend on these sectors [23]. For instance, the 1995 drought caused a decline in rice and wheat production of 3.5 × 10^6^ tons [24]. In a recent study, Hoque et al. [23] reported that the north-western region of Bangladesh is very vulnerable to droughts, which may affect the agriculture, forests, fisherires and other livelihood options in the future unless the adaptive and mitigation measures are not taken place. Thus, understanding and assessment of drought conditions in such drought-prone regions is of high importance for decision making and for management practices.

Assessment of drought can be done based on the nature of water scarcities, mean periods, level of truncation, and regionalization methods [25,26,27]. In general, drought is regarded as an insidious hazard since its effects are typically detected very slowly over an extended period and it is difficult to mitigate their effects [28]. In addition, the assessment of drought indices is most effective for specific locations, whereas the lack of appropriate geographic information and dependable data is regarded as the primary challenge to climate-data-based drought assessment [29,30]. However, a growing body of evidence has reported that several vegetation indices are suitable for assessing drought conditions in a particular area for a given timeframe [31,32,33,34]. For example, Orimoloye et al. [35] stated that drought can be evaluated by analyzing green vegetation through Normalized Vegetation Water Index (NDWI), Soil Adjusted Total Vegetation Index (SATVI), Land Surface Temperature (LST), Normalized Difference Vegetation Index (NDVI), Normalized Drought Dryness Index (NDDI), Vegetation Condition Index (VCI) and Temperature Vegetation Dryness Index (TVDI). Furthermore, satellite-based remote sensing data provides an advanced way to understand the scenario of natural resource depletion [36], monitor ecosystem health [37], and predict the climate-induced changes in soil, water, and biodiversity [38,39].

Multispectral remote sensing is an excellent method for evaluating drought and vegetation dynamics [40]. Suitable indices (e.g., NDVI and NDWI) of satellite-derived remote sensing data (e.g., Landsat) can be used to assess the impacts and severity of drought. The vegetation indices such as NDWI, SAVI, LST, and NDVI can be derived from the reflectance of green vegetation-associated blue, green, near-infrared, and red spectral bands [35]. Despite the great efforts that have been made in recent years to assess drought occurrence, drought risk reduction, and drought adaptation across many regions (including north-western region) of Bangladesh [19,33,41,42], the evidence of drought assessment using multiple vegetation indices in the north-western region of Bangladesh is limited [43]. An advanced understanding of the land use and land cover change (LULC), and vegetation greenness-drought relationship using multiple vegetation indices is required to provide compelling evidence of the occurrence and severity of drought. Utilizing remote sensing data and GIS tool, this study was undertaken to (i) examine the LULC changes for every 5 year interval during 1990–2020, (ii) estimate the severity of drought employing several vegetation indices and (iii) identify the drought occurrences in the north-western region of Bangladesh for the period 1990–2020.

The originality of this study lies in the application of five different vegetation indices of recent 3 decades for assessing the changes of LULC and examining the occurrences and severity of drought in a drought-prone semi-arid region of Bangladesh, which provides a robust understanding of the spatiotemporal changes of LULC and an evidence of the consistency of the application of remote sensing-based indices in drought monitoring.

## 2 Materials and methods

### 2.1 Study area

**Fig. 1:**
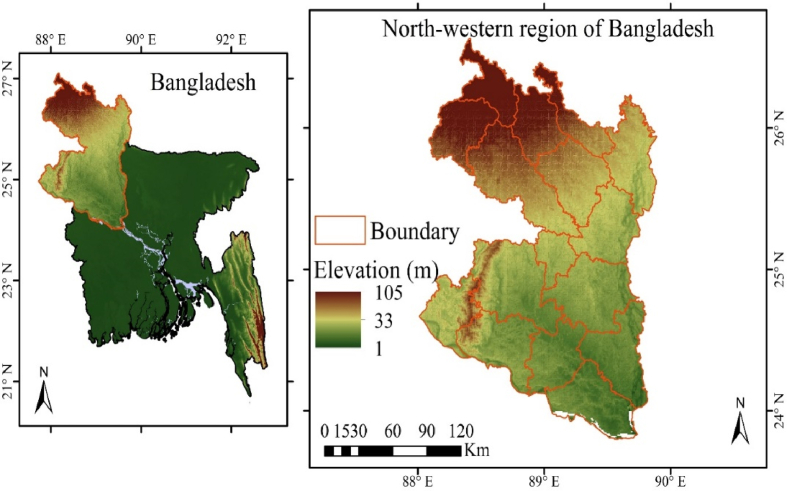
The location of the study area (north-western region) in Bangladesh.

The north-western region of Bangladesh was considered as the study area to conduct this study (Fig. 1). The north-western region includes Rangpur and Rajshahi divisions and the total area of this region is 32,000 km^2^ [44]. This region is bound by the Ganges and Jamuna rivers within the south and east, and both north and west side bound by the Indian border. There are 8 districts (Kurigram, Rangpur, Lalmonirhat, Gaibandha, Dinajpur, Panchgarh, Thakurgaon, and Nilphamari) in Rangpur divisions and 8 districts (Rajshahi, Natore, Nogaon, Chapainawabganj, Joypurhat, Pabna, Bogra, and Sirajganj) in Rajshahi division [45].

As the region is situated over tropic of cancer, the north-western region is influenced by strong anomalies in temperature and precipitation. This region usually experiences a typical monsoon climate, where considerable amount of the precipitation falls between May and September (1583 mm). Besides, maximum and minimum temperature varies from 35 °C to 25 °C and from 20 °C to 10 °C, respectively. The humidity remains high in the monsoon, while drops significantly at the end of dry season [46,47]. There are three seasons in this region: i) dry winter (December–February), ii) pre-monsoon hot summer (March–May), and iii) rainy monsoon (June–October).

Geographically the study area is divided into four regions including i) Barind tract, ii) Himalayan piedmont plains, iii) alluvial lowland along with the R.B-Jamuna, and iv) alluvial lowland along the River Ganges [48]. About 59% of the agriculture land of this area is under the irrigation project and about 75% of irrigation water gets from the surface water. But during dry season, groundwater is the single source of irrigation in this region [49]. Soil of this region becomes dry because of limited and untimely precipitation and the obstacles in the flow of the river [46,50].

### 2.2 Data collection

**Fig. 2:**
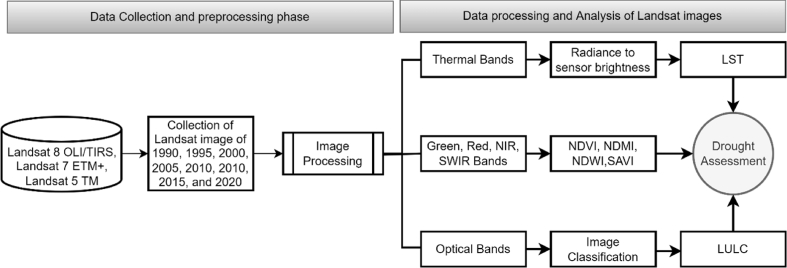
Conceptual framework of the study.

The study comprises multi-stage analysis to exact the drought conditions over the recent 3 decades (1990–2020) at five years interval (i.e., 1990, 1995, …, and 2020) in the north-western region of Bangladesh. The conceptual framework of the present study was presented in Fig. 2. The individual satellite images were collected for a five-year interval from 1990 to 2020. Twenty-eight sets of Landsat images from four different paths and rows were extracted from the USGS archive (https://earthexplorer.usgs.gov/). Four sets of images were required to cover the full study area. The details of the data collected from the USGS archive for the years 1990, 1995, 2000, 2005, 2010, 2015, and 2020 are presented in Table S1. The images for the study were processed to Level one precision and terrain corrected product (L1TP). The Landsat data: Thematic Mapper (TM), Enhanced Thematic Mapper (ETM+), and Thermal Infrared Sensor (TIRS) were collected from the USGS. The study area is covered by 4 Landsat images which are row 42 and 43 of path 138 and row 42 and 43 of path 139. The Universal Transverse Mercator (UTM) within zone 46 north and WGS-1984 datum system was applied in the Landsat data.

### 2.3 Image processing

We used ArcGIS 10.8 for the analysis of the Landsat images. Except the thermal and panchromatic bands, individual bands were combined by ‘Layer stacking’ process, as the thermal and panchromatic bands have different spatial resolutions and images were combined by ‘Mosaic’ process. The ‘Mask’ process extracted area of interest. Sensor-specific information was used for calibration and the relevant sensor data were collected from metadata files of the Landsat images. For the radiometric calibration, the DN values were converted to at-sensor radiance and then Top of Atmosphere (TOA) reflectance. Finally, the atmospheric correction was made to remove dark objects.

### 2.4 Indices to assess the drought condition

To assess the drought condition, 5 indices were analyzed. These are: i) Land Surface Temperature (LST), ii) Normalized Difference Vegetation Index (NDVI), iii) Normalized Difference Water Index (NDWI), iv) Normalized Difference Moisture Index (NDMI) and v) Soil Adjusted Vegetation Index (SAVI). The indices used for assessing the drought condition of the study area are presented in Table 1.

**Table 1:** Vegetation Indices (normalized difference vegetation index (NDVI), normalized difference water index (NDWI), normalized difference moisture index (NDMI) and soil adjusted vegetation index (SAVI)), and land surface temperature (LST) used for the assessment of drought conditions.

No.	Index	Equation	Reference
1	LST	$LST=\frac{Tb}{1+\left(\lambda *Tb\left(Ƿ\right)\right)\mathrm{Ln}€}$	[35]
2	NDVI	$NDVI=\frac{ǷNIR-ǷNIR}{ǷNIR+Ƿred}$	[51]
3	NDWI	$NDWI=\frac{ǷGreen-ǷNIR}{ǷGreen+ǷNIR}$	[52]
4	NDMI	$NDMI=\frac{ǷNIR-ǷSWIR}{ǷNIR+ǷSWIR}$	[53]
5	SAVI	$SAVI=\frac{ǷNIR-ǷNIR}{ǷNIR+Ƿred+L}(1+L$)	[54]

**NDVI:** The NDVI is relevant to the proportion of radiation, which is absorbed by photosynthesis process. The NDVI assesses the amount and strength of vegetation on ground. The level of NDVI is associated to the extent of photosynthetic process in the experimental vegetation. Generally, greater values of NDVI depict the larger strength and quantities of vegetation. Here, NDVI provides a sign of area on how much it covers green vegetation at a specific location on the earth. The calculation of NDVI is given in equation (i) [55].

(i) $$NDVI=\frac{ǷNIR-Ƿred}{ǷNIR+Ƿred}$$

where, Near Infrared (NIR) and Red light (RED) are the reflectance in the near-infrared and red bands, respectively. The values of NDVI range between −1 and +1. High NDVI value represents healthy green vegetation because NIR light reflects more than red light.

**SAVI:** The SAVI calculation is almost the same as the NDVI. In the calculation of the SAVI, a soil adjustment factor is used for reducing the external effect. The calculation of SAVI is given in equation (ii).

(ii) $$SAVI=\frac{ǷNIR-Ƿred}{ǷNIR+Ƿred+L}\left(1+L\right)$$

where Ƿ is the reflectance in NIR and red band and L is the soil adjustment factor. The optimal value for the soil adjustment factor is L = 0.5 [56].

**NDWI:** The NDWI is calculated by the subtraction of green and near-infrared bands then dividing by the sum of the green and near-infrared bands [57]. The equation (iii) represents the procedure for estimation of NDWI.

(iii) $$NDWI=\frac{ǷGreen-ǷNIR}{ǷGreen+ǷNIR}$$

where Ƿ is the reflectance in the Green and near-infrared band.

**NDMI:** The NDMI is calculated from the NIR and the shortwave infrared (SWIR) band. In Landsat 8, the NIR is the band-5 and the SWIR is the band-6. For the Landsat TM or ETM+ the NIR and SWIR are consequently band-4 and band-5 [58]. The equation (iv) shows the procedure for calculation of NDMI.

(iv) $$NDMI=\frac{ǷNIR-ǷSWIR}{ǷNIR+ǷSWIR}$$

### 2.5 Deriving land surface temperature (LST)

**LST from Landsat TM and ETM** + **Images:** The LST from the Landsat TM and ETM + data were obtained in two steps. First, the DN values were transformed to spectral radiance applying the spectral radiance scaling approach (equation v) [59].

(v) $$L\lambda =\frac{\left(LMAX-LMIN\right)}{\left(QCALMAX-QCALMIN\right)}*\left(\left(DN-QCALMIN\right)+LMIN\right)$$

where, LMAX = Spectral Radiance Scaled to QCALMAX in W/(m^2^*sr*μm), LMIN = Spectral Radiance Scaled to QCALMIN in W/(m^2^*sr*μm), QCALMAX = Maximum Quantized Calibrated Pixel Value (Corresponding to LMAX) in DN = 255, QCALMIN = Minimum Quantized Calibrated Pixel Value (Corresponding to LMIN) in DN = 1.

In the second step, the radiance was then converted to degree kelvin by the following formula in equation (vi)

(vi) $$TB=\frac{\frac{K2}{\ln\left(K1\right)}}{L\lambda +1}$$

where TB = Sensor Brightness Temperature in Degree Kelvin, Lλ = Spectral radiance at-sensor in W/(m^2^*sr*μm), K1 = 1260.56 k, K2 = 607.66 (Wm^−2^sr^−1^μm^−1^).

**LST from the Landsat OLI/TIRS:** For deriving LST from OLI/TIRS data, a two-step process has been taken. At the first step, the bands were converted into TOA spectral radiance using metadata file (equation vii) [59].

(vii) $$L\lambda =ML*Q_{cal}+AL$$

where, ML and AL are reflectance coefficients and Lλ = TOA spectral radiance (W/m^2^*sr*μm), Qcal = Pixel value (DN).

In the second step, the Brightness Temperature (T_B_) has been calculated by using the formula in equation (vii).

(viii) $$TB=\frac{\frac{K2}{\ln\left(K1\right)}}{L\lambda +1}$$

Where T_B_ is the at sensor Brightness temperature in degree kelvin, Lλ = Spectral radiance at-sensor in W/(m^2^*sr*μm), K1 = 774.8853 K and K2 = 1321.789 (Wm^−2^sr^−1^μm^−1^).

### 2.6 Validation of the results

There are several methods to validate the remote sensing results. In this study, Landsat ETM + NDVI data was used for the validation of the results, which can provide an accurate representation of the earth surface. This technique was also used in previous studies in the same region [43,60] and the results were consistent with the present study. The validation of other indices has been confirmed in recent studies (LST [61], NDWI [62], NDMI [63] and SAVI [64]). The validation results of previous studies indicate that these remote sensing data can be used for hydrological approaches such as drought monitoring.

### 2.7 Data analysis

The temporal changes of area of the five LULC was assessed using the Kendall's correlation coefficient [65]. The relationships between NDVI and LST for the seven study period were obtained using Pearson's correlation [66]. Here, the dependent variable was NDVI and the independent variable was LST. We used a linear model to estimate the trend [67], and the R^2^ and p values of correlation coefficient have been shown using the ‘stat.cor’ function in the ggplot package in R. All statistical analyses were performed in the statistical package R, version 4.0.3 [68].

## 3 Results

### 3.1 Changes in land use and land cover (LULC) between 1990 and 2020

**Fig. 3:**
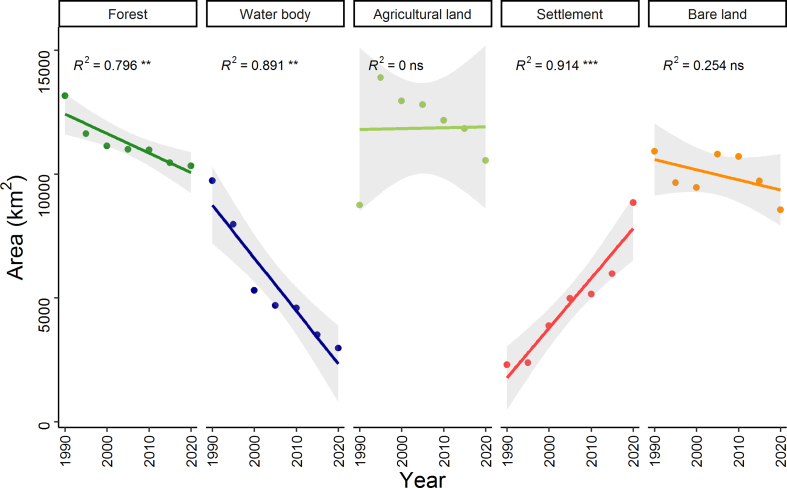
Temporal changes of five Land Use and Land Cover (LULC: forests, water bodies, agricultural land, settlement and bare land) area (km^2^) in the north-western region of Bangladesh for the period 1990–2020. Solid lines indicate linear regressions of the LULC area were increased or decreased over the three decades. Bands near the lines represent 95% confidence intervals of changes in the coverage. The *R*^2^ values of the correlation coefficient of are shown. Asterisk (*, ** and ***) indicates the significance of the changes of the LULC area at *p* < 0.05, <0.01, and <0.001.

**Fig. 4:**
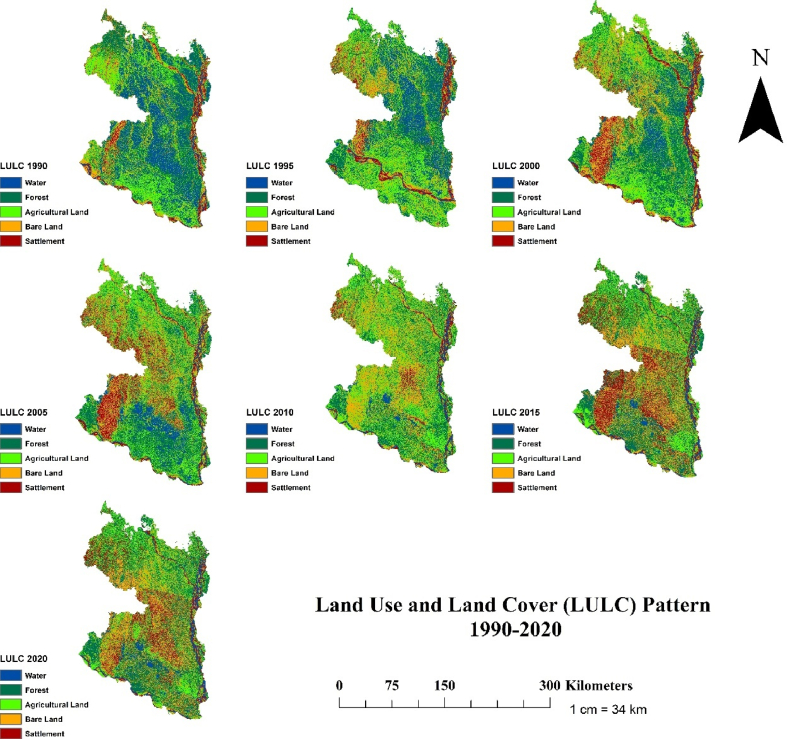
Land Use and Land Cover (LULC: water body, forests, agricultural land, bare land and settlement) pattern between 1990 and 2020 in the north-western region of Bangladesh.

The analysis of the unsupervised classification of the study area and the corresponding land cover area have been presented in Figs. 3 and 4, respectively, for the 7 periods (every 5 years). Results shows that the coverage of forests, and water bodies were significantly decreased (forests: *R*^*2*^ = 0.796, *p* < 0.01; water bodies: *R*^*2*^ = 0.891, *p* < 0.01), while the settlement was expanded (*R*^*2*^ = 0.914, *p* < 0.001) over the 30 years period (Figs. 3 and 4). For instance, the area of the water bodies declined threefold from 9738 km^2^ in 1990–3509 km^2^ in 2020. Similarly, the forests coverage decreased from 13,171 km^2^ in 1990 to 10,339 km^2^ in 2020 (Fig. 3). The large decrease in forests, water bodies and bare land mostly resulted from the rapid expansion of settlement (5.1% in 1990 to 21.45% in 2020) and agricultural land (8552 km^2^ in 1990 to 10,553 km^2^ in 2020). More than 2370 km^2^ area of bare land was converted to other LULCs, but the changing trend was not significant over the study period (*R*^*2*^ = 0.254, *p* > 0.05).

The decreasing trend of forests and water bodies and increasing trend of settlement and agricultural land in our study is consistent with some recent studies in the northern region of Bangladesh [69,70,71]. One obvious changing trajectory of LULC has resulted from the expansion of the built-area to accommodate the growing population, which is consistent with Samad et al. [71], who reported that urban expansion in the northern region of Bangladesh decreased the forests and water bodies. The expansion of settlement may be attributed to population migration to cities in pursuit of better standard of living and higher income. Despite the increasing rate of population migration from the rural to urban areas across the countries (e.g., around 3 million people are anticipated to relocate into urban areas every week worldwide) [72], population migration in Bangladesh, especially from resource-scarce areas to urban areas is even high due to multifaceted reasons. For example, in a study of migration dynamics of mega-delta cities in Asia and Africa, Seto [73] showed that massive influx of urban population in the Ganges-Brahmaputra delta was caused by socio-economic disparities between rural and urban areas.

### 3.2 Spatiotemporal patterns of vegetation indices-based drought conditions

**Fig. 5:**
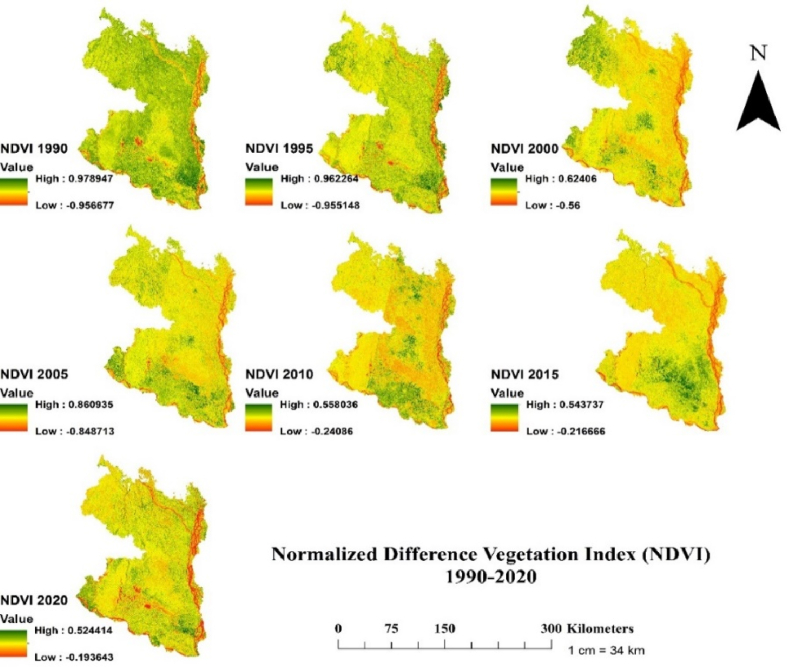
Temporal changes of Normalized Difference Vegetation Index (NDVI) in the north-western region of Bangladesh at 5 years interval for the period 1990–2020.

**Fig. 6:**
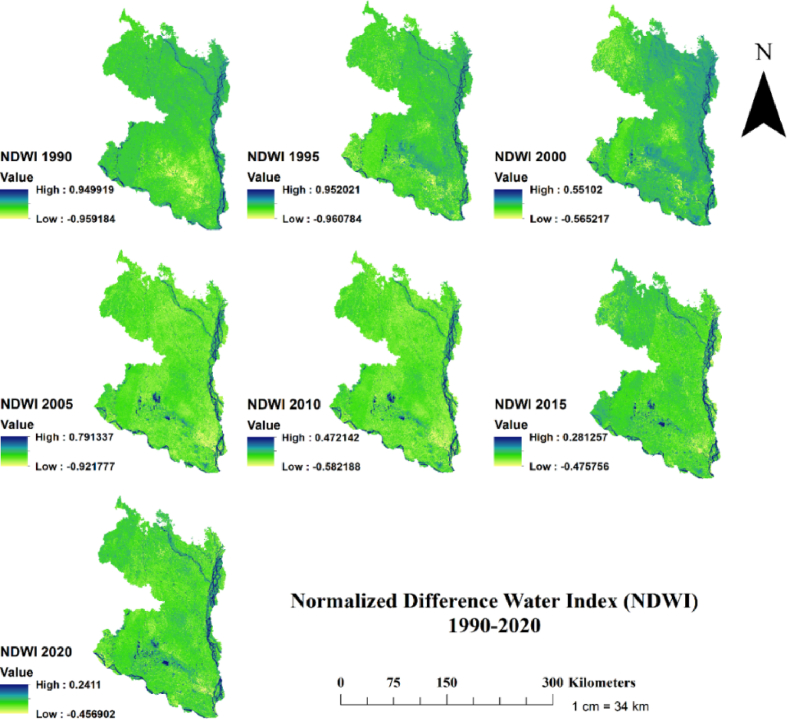
Temporal changes of Normalized Difference Water Index (NDWI) in the north-western region of Bangladesh at 5 years interval for the period 1990–2020.

**Fig. 7:**
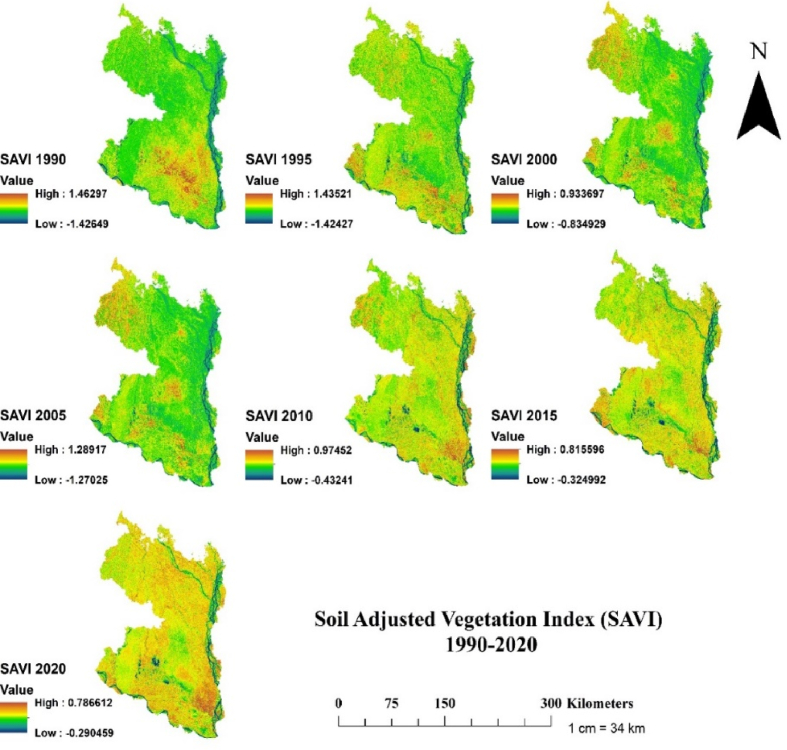
Temporal changes of Soil Adjusted Vegetation Index (SAVI) in the north-western region of Bangladesh at 5 years interval for the period 1990–2020.

**Fig. 8:**
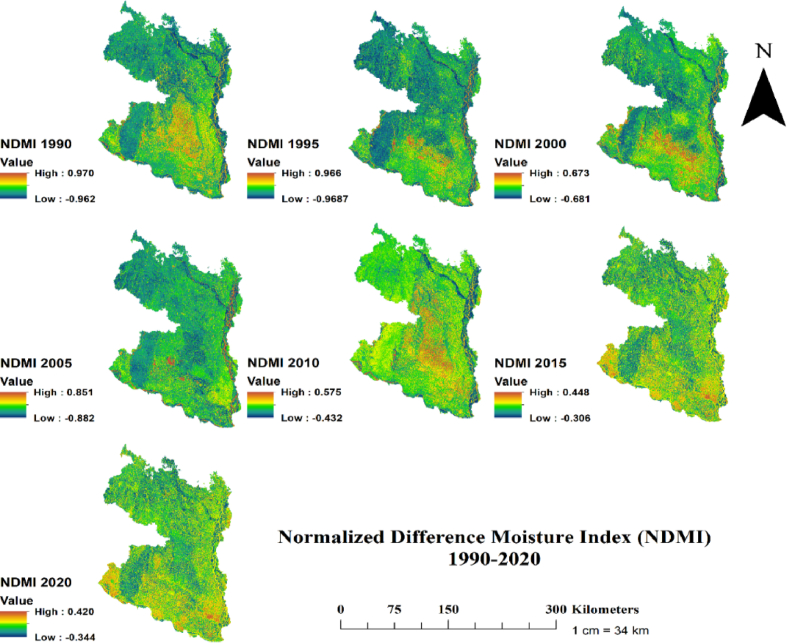
Temporal changes of Normalized Difference Moisture Index (NDMI) in the north-western region of Bangladesh at 5 years interval for the period 1990–2020.

In attempting to understand the spatiotemporal changes of drought conditions in the north-western region of Bangladesh, four vegetation indices (NDVI, NDWI, SAVI and NDMI) were considered (Figs. 5–8). First, for NDVI index, the highest value of NDVI was 0.978, 0.962, 0.624, 0.860, 0.550, 0.543 and 0.524 for the year 1990, 1995, 2000, 2005, 2010, 2015 and 2020, respectively (Fig. 5). The 30-year analysis of NDVI value has a negative trend which means the vegetation of the study area is being stressed due to the increasing trend of drought events as asserted by previous investigation [62]. The NDVI value was highest in 1990 and lowest in 2020. The driest and wettest conditions were recorded in 2020 and 1990 in the study area (Fig. 5). The decreasing vegetation NDVI value could be attributed to increasing drought intensity and frequency in the study area, which supports the notion that drought-induced stress slows down the photosynthetic process [37], enhances mortality, and reduces plant recruitment and seedling establishment. A recent study in the north-western region of Bangladesh reported an increasing drought impact on the existing water courses (e.g. river and canal) [74], which further led the lowering of ecosystem functioning [43]. The higher NDVI values in 2005 compared to 2000 in our study could be resulted from the large scale afforestation in degraded land and participatory forestry initiatives by the government and implementation of some donor-funded afforestation projects (e.g., community forestry project in north-western region of Bangladesh funded by Asian Development Bank [75]. For example, with the support of World Food Programme, Bangladesh government implemented social forestry programme during 1990–1998. Nearly 31 million trees were planted during this period across the country, of which the main targeted areas was the degraded and fallen lands. Due to deteriorating land and lower socio-economic conditions, the north-western region of the country received more attention from this programme. However, there could be other reasons of such discrepancy (e.g., occurrence of drought in 2000), which is evident in other studies [21]. For example, an exceptional drought was recorded in the north-western region in 2000, which adversely reduced the agriculture productivity and increased the tree mortality [21].

Second, when NDWI was considered, the highest NDWI values showed a decreasing trend over the study period (Fig. 6). For instance, the highest NDWI value in 1990 was 0.949 and in 2020 was 0.241. The decreasing trend of NDWI indicates that the north-western region of Bangladesh is facing water shortage and experiencing increasing intensity and frequency of drought events, as it is known that regions with low NDWI values are more prone severe drought, whereas regions with high NDWI values indicate low or no drought [62,76,77]. Our findings on lowering NDWI values in the recent decade could be attributed to lower precipitation and increasing annual temperature, which is consistent with Rahaman et al. [24], who reported that annual average precipitation in the north-western region of Bangladesh decreased from 151.50 mm in 1994–2003 to 138.09 mm in 2004–2013. This lowering precipitation and increasing annual temperature in our studied regions [24] could negatively affect NDWI values. The evidence of lower precipitation and reduction of groundwater recharge in the north-western region of Bangladesh has recently been reported by Siddik et al. [78], which showed that groundwater recharge decreases from 2.6 mm/year in the basin scale to 17.1 mm/year in regional level due to changes of LULC for the period 2006–2016.

**Fig. 9:**
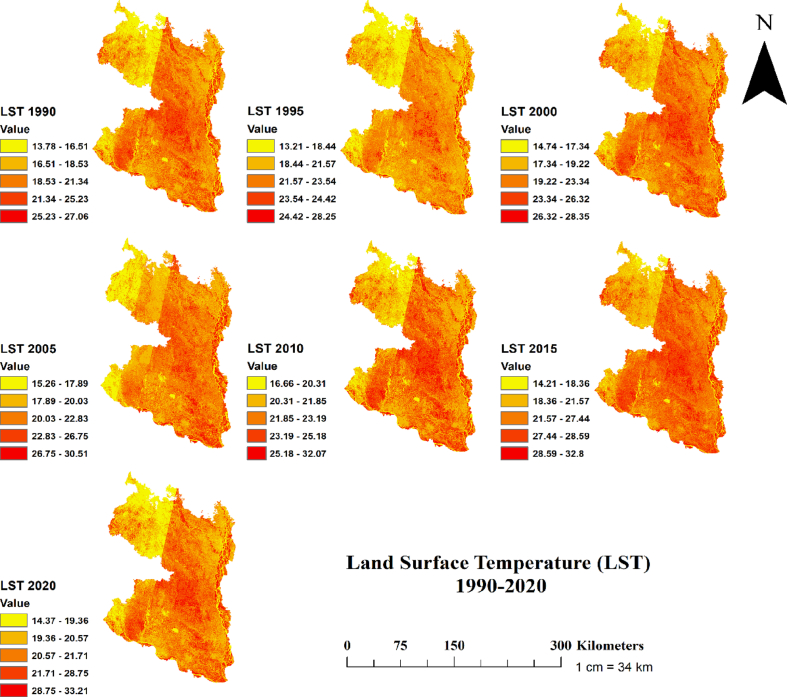
Temporal changes of Land Surface Temperature (LST) in the north-western region of Bangladesh at 5 years interval for the period 19,902,020.

**Fig. 10:**
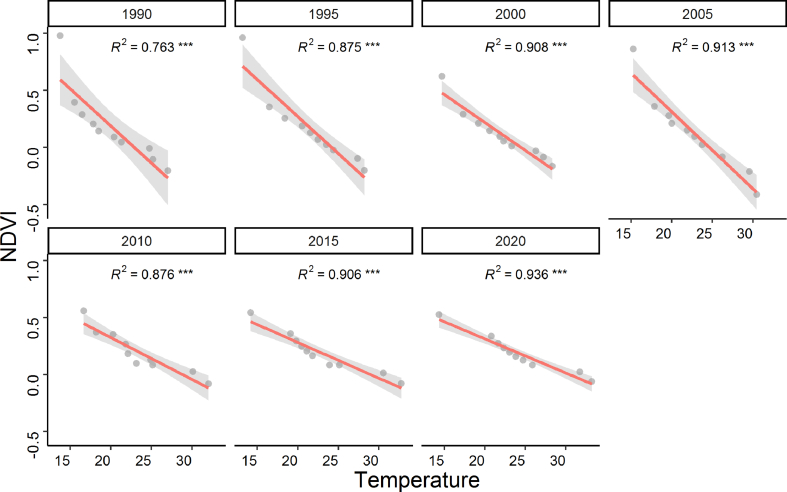
Relationship between the Normalized Difference Vegetation Index (NDVI) and Land Surface Temperature (LST) in the north-western region of Bangladesh at 5 years interval for the period 1990–2020. Solid lines indicate linear regressions of the decreased NDVI with increasing LST for the year 1990, 1995, 2000, 2005, 2010, 2015 and 2020. Bands near the lines represent 95% confidence intervals of changes in the NDVI. The *R*^2^ values of the correlation coefficient of are shown. Asterisk (***) indicates the significance of the changes of the NDVI at *p* < 0.001.

Finally, examination of SAVI and NDMI indices revealed that the highest SAVI and NDMI values were in decreasing trend over the three decades (Figs. 7 and 8). For instance, the highest SAVI value declined two times over the study period, from 1.462 in 1990 to 0.787 in 2020 (Fig. 7). Likewise, the highest NDMI value in 1990 was 0.97 and in 2020 was 0.42 (Fig. 8). Consistent with lowering NDVI and NDWI values, the decreasing SAVI and NDMI values over the study period might be linked to increasing climatic variability. For instance, using the effective drought index, a recent study showed that the northern region of Bangladesh has been experiencing frequent seasonal and annual droughts [18]. As the values of all indices were found decreasing pattern over the three decades, we further assessed the trends of land surface temperature (LST) and correlated with NDVI to examine whether the lowering indices values are driven by LST, which is shown in section 3.3 (Figs. 9 and 10).

### 3.3 Spatial pattern of land surface temperature (LST) and its relationship with NDVI

Examination of LST showed an increasing trend for the period 1990–2020 (Fig. 9). This increasing temperature had severe impacts on vegetation, as we found significant negative correlation between NDVI and temperature for all 7 studied years (Fig. 10; all *p* < 0.001; *R*^*2*^ = 0.763 for the year 1990, *R*^*2*^ = 0.875 for 1995, *R*^*2*^ = 0.908 for 2000, *R*^*2*^ = 0.913 for 2005, *R*^*2*^ = 0.876 for 2010, *R*^*2*^ = 0.906 for 2015, *R*^*2*^ = 0.936 for 2020). The negative relationship between vegetation NDVI and LST in our study could be resulted from several mechanisms. One mechanism is that elevated temperature enhances the rate of soil moisture depletion, impairs plant photosynthesis, reduces plant recruitment and seedling establishment, and increases mortality [79,80,81]. Another possible mechanism could be caused from lower resistance and resilience of vegetation due to drought memory effects [[82], [83], [84], [85]]. Moreover, the changes in LULC in the northern region can also influence the NDVI, which has been reported in previous studies [43,70,71]. Increasing land surface temperature highlights that the north-western region of Bangladesh is facing climate-induced disturbances (e.g. droughts and heat waves). The negative relationship between NDVI and LST suggests that vegetation in our studied region suffered from drought-induced stresses.

## 4 Conclusion

Drought is a global problem that can have catastrophic impacts on the lives and livelihoods, including agriculture and forests, especially in arid regions. The arid climate in the north-western region of Bangladesh is mostly attributed to the country's topography, which makes this region vulnerable to droughts. Using multiple vegetation and climate indices (NDVI, NDWI, SAVI, NDMI and LST) derived from Landsat, this study represents a spatially synergistic way to assess the occurrence of drought in the north-western region of Bangladesh from 1990 to 2020 at five-year interval. The LULC analysis was performed using five land use features namely water bodies, forests, agricultural land, bare land and settlement. The decreasing trend of forests and water bodies mostly resulted from the expansion of settlement and agricultural land. Although temporal analysis of the values of all indices (i.e., NDVI, NDWI, SAVI and NDMI) showed a decreasing trend over the study period, the recent decade (2010–2020) exhibited higher decreasing rate than that in previous decades. We also found that the vegetation area was negatively correlated with LST and NDVI, while the settlement and bare land exhibited a positive relationship with LST and NDVI. These findings indicate that the vegetation abundance is directly correlated with the drought condition. The downward trend forest NDVI value over the last 3 decades highlights that increasing LST accelerated the frequency and intensity of droughts, which enhanced plant mortality and inhibited plant recruitment and seedling establishment. Our study provides evidence of temporal changes in LULC, drought occurrence and its impact on vegetation health, which is of practical implication in future land use planning and ecosystem restoration interventions.

## Declarations

### Author contribution statement

Ashim C. Das; Shihab A. Shahriar; Mohammed Abdus Salam: Conceived and designed the experiments. Ashim C. Das; Shihab A. Shahriar; Md A. Chowdhury; Shahed Mahmud, Md. Kamruzzaman Tusar; Mohammed Abdus Salam: Performed the experiments. Ashim C. Das; Md Lokman Hossain; Mohammed Abdus Salam: Analyzed and interpreted the data. Md A. Chowdhury; Shahed Mahmud; Md Kamruzzaman Tusar; Romel Ahmed; Mohammed Abdus Salam: Contributed reagents, materials, analysis tools or data. Ashim C. Das; Md Lokman Hossain; Romel Ahmed; Mohammed Abdus Salam: Wrote the paper.

### Funding statement

Dr. Mohammed Abdus Salam was supported by Research Cell, Noakhali Science and Technology University [NSTU/RC-ESDM/T-21/56].

### Data availability statement

Data associated with this study has been deposited at https://earthexplorer.usgs.gov/

### Declaration of interest's statement

The authors declare no competing interests.

### Additional information

The article contains one supplementary material.
