# Supplementary material for: Assessment of remote sensing-based indices for drought monitoring in the north-western region of Bangladesh
Source: Heliyon. 2023 Jan 21;9(2):e13016. doi: 10.1016/j.heliyon.2023.e13016 (PMC9900510; doi:10.1016/j.heliyon.2023.e13016)
Supplement: Multimedia component 1 [file mmc1.pdf]

Supplementary Information for

Assessment of remote sensing-based indices for drought monitoring in the North-western region of Bangladesh

Ashim C. Das[1], Shihab A. Shahriar[1,2], Md. A. Chowdhury[3], Md. Lokman Hossain[4,5], Shahed Mahmud[1], Md. Kamruzzaman Tusar[1], Romel Ahmed[6], Mohammed Abdus Salam[1*]

[1]Department of Environmental Science and Disaster Management, Noakhali Science and Technology University, Noakhali 3814, Bangladesh

[2]Department of Earth and Atmospheric Sciences University of Houston, TX 77004, USA

[3]Department of Climate and Disaster Management, Jashore University of Science and Technology, Jashore- 7408, Bangladesh

[4]Department of Environment Protection Technology, German University Bangladesh, Gazipur, Bangladesh

[5]Department of Geography, Hong Kong Baptist University, Hong Kong, China

[6]Department of Forestry and Environmental Science, Shahjalal University of Science and Technology, Sylhet 3114, Bangladesh

*Corresponding Author

Mohammed Abdus Salam (E-mail: masalam.esdm@nstu.edu.bd);

Telephone: +8801917635348

Table S1: Detailed information of the collected satellite data.

Year	Acquisition Date	Path/Row	Landsat	Sensor	Number of Bands	Cell Size	Data Format
	14/11/1990	138-42					
	14/11/1990	138-43					
1990	23/10/1990	139-42	5	TM	7	30	GeoTiff
	23/10/1990	139-43					
	28/10/1995	138-42					
	28/10/1995	138-43					
1995	13/10/1995	139-42	5	TM	7	30	GeoTiff
	12/10/1995	139-43					
	18/10/2000	138-42					
	7/10/2000	138-43					
2000	13/11/2000	139-42	5	TM	7	30	GeoTiff
	16/11/2000	139-43					
	07/11/2005	138-42					
	08/11/2005	138-43					
2005	14/11/2005	139-42	5	TM	7	30	GeoTiff
	14/11/2005	139-43					
	05/11/2010	138-42					
	11/10/2010	138-43					
2010	12/11/2010	139-42	8	OLI/TIRS	11	30	GeoTiff
	14/11/2010	139-43					
	17/10/2015	138-42					
	18/10/2015	138-43					
2015	26/11/2015	139-42	8	OLI/TIRS	11	15,30	GeoTiff
	25/11/2015	139-43					
	02/10/2020	138-42					
	02/10/2020	138-43					
2020	23/11/2020	139-42	8	OLI/TIRS	11	15,30	GeoTiff
	23/11/2020	139-43					
